# Secondary caries in fixed dental prostheses: Long‐term clinical evaluation

**DOI:** 10.1002/cre2.696

**Published:** 2022-11-26

**Authors:** Ali Alenezi, Omar Alkhudhayri, Fahad Altowaijri, Lina Aloufi, Fatimah Alharbi, Maha Alrasheed, Hind Almutairi, Abdulltif Alanazi, Mohammed Yehya, Dhafer Al Asmari

**Affiliations:** ^1^ Department of Prosthodontics, College of Dentistry Qassim University Buraydah Saudi Arabia; ^2^ Interns' Clinics, College of Dentistry Qassim University Buraydah Saudi Arabia; ^3^ Department of Restorative Dentistry Prince Sultan Military Medical City Riyadh Saudi Arabia; ^4^ Department of Periodontology, College of Dentistry Qassim University Buraydah Saudi Arabia

**Keywords:** fixed dental prosthesis, oral hygiene, plaque index, secondary caries

## Abstract

**Objectives:**

Even with excellent fixed dental prostheses (FDPs), there will be a substantial risk of biological complications, such as caries, if proper oral hygiene is not maintained. This study aimed to evaluate the risks of developing secondary caries with FDPs in relation to the patient oral hygiene status.

**Material and Methods:**

Clinical and radiographic examinations were performed for patients to collect data related to their FDP complications and oral hygiene status. The main clinical parameter analyzed was secondary caries. Complications such as a fracture, debonding, or the need for endodontic therapy were also analyzed. The interval survival rate and the cumulative survival rate of FDPs from the time of treatment to the time of follow‐up were analyzed.

**Results:**

A total of 423 patients (with a total of 1116 FDPs) were examined in this study, with a mean age of 43.7 years and a mean follow‐up time of 7 years. Regarding complications, secondary caries was detected in 94 FDPs (8.4%), fracture (or chipping) in 85 (7.6%) cases, need for endodontic treatment in 42 cases (3.7%), and debonding in four (0.3%) cases. Secondary caries was found in nine out of 219 FDPs (4%) in patients with good oral hygiene, 35 out of 634 FDPs (5.5%) in patients with fair oral hygiene, and 50 out of 272 FDPs (18.4%) in patients with poor oral hygiene (*p* ≤ .001).

**Conclusions:**

Good oral hygiene has a great influence on reducing the risk of secondary caries in patients with FDPs. The occurrence of secondary caries is a common complication in patients with poor oral hygiene.

## 1 INTRODUCTION

Today, dental treatment through fixed dental prostheses (FDPs) helps millions of patients by controlling oral disease and restoring mouth function and aesthetics. Due to the time and costs associated with this type of treatment, patients expect to receive treatment that is successful, durable, and survives for a long period of time under normal conditions (Glantz et al., 2002). Many reports have confirmed that FDPs have a long survival rate of up to 20 years (De Backer et al., 2006, 2008). Failure of FDPs can be the result of various mechanical and biological complications (Sailer et al., 2006; Solá‐Ruiz et al., 2022). Although there is no standardized definition of FDP failure, the need to replace an existing FDP or to extract a tooth may be seen as a clear indication of failed treatment (Heintze & Rousson, 2010; Scurria et al., 1998). For instance, a common failure seen with this type of treatment is when an abutment tooth needs to be extracted due to biological complications or is associated with an irreparable problem (Guess et al., 2014). Some published studies have not considered certain complications, such as chipping or cracks in a crown, as failures because they are repairable issues, particularly when the defects are small (Rinke et al., 2018).

Most investigations have focused on evaluating mechanical complications with FDPs (Heintze & Rousson, 2010). This is understandable, as these types of complications, such as a fracture or debonding, are believed to be the most common type of issue associated with FDPs (Alenezi et al., 2021; Pjetursson et al., 2015). Many clinicians believe that these mechanical complications may have little effect on the prognosis of abutment teeth, as they can be restored again (Maroulakos et al., 2019). On the other hand, some of the biological complications associated with FDPs, such as caries or the need for endodontic treatment, carry a great risk to the long‐term prognosis of restorations (Basnyat et al., 2015; Srimaneepong et al., 2022).

The incidence of dental caries has been linked with poor oral hygiene, which may allow plaque to accumulate on tooth surfaces (Taraszkiewicz‐Sulik et al., 2012). With natural dentition, an intact tooth surface undergoes a self‐cleaning process with the help of saliva that regulates pH and balances oral microflora (Mattos‐Graner et al., 2014). For patients with FDPs, this self‐cleaning process can be limited due to various factors, such as the presence of a connector with a dental bridge or gaps at the tooth–restoration interface (Kois, 1996). In these cases, dental plaque can accumulate easily in different places around the FDPs, such as the crown margins underneath the bridge connector or between the bridge pontic and the oral mucosa (Taraszkiewicz‐Sulik et al., 2012). Thus, patients with FDPs should be informed of the importance of oral hygiene and should be aware of the oral cleaning aids suitable for their restorations. In addition to oral hygiene, successful FDPs should be made with a proper crown margin and morphology that will allow good integration into dental hard tissues and easier oral care. Many reports have revealed that the marginal adaptation of FDPs has a great effect on the longevity of dental restorations (Contrepois et al., 2013; Heboyan, 2019). Large marginal gaps can expose the luting cement to oral fluid, which can facilitate microleakage and the breakdown of the luting agent. This subsequently allows food accumulation and plaque retention, which may lead to caries and periodontitis (Beschnidt & Strub, 1999).

Even with excellent FDPs, there will be a substantial risk of biological complications, such as caries, if proper oral hygiene is not maintained (Srimaneepong et al., 2022). Patients should be made aware of how daily oral care can impact their dental treatment prognoses. The aim of this study was to investigate the long‐term biological and mechanical complications associated with FDPs. It also aimed to evaluate the risks of developing secondary caries with FDPs in relation to the patient oral hygiene status. The null hypothesis was that patients with different oral hygiene statuses would have the same risk of developing caries lesions with their FDPs.

## 2 MATERIAL AND METHODS

This study received ethical approval from the ethical committee at Qassim University to evaluate oral hygiene and the condition of previously cemented FDPs (crowns, veneers, and bridges). The authors examined patients who visited the dental clinics at the College of Dentistry at Qassim University between July 2020 and May 2021. The study involved only patients who were above 18 years of age and were able to provide signed informed consent. All evaluated FDPs should be entirely tooth‐supported to be included in this study. Implant‐supported FDPs or removable prostheses were excluded. Patients' anonymity was maintained throughout the study. Clinical and radiographic examinations were performed for all patients to collect data related to their FDP complications and oral hygiene status. The main clinical parameter analyzed was secondary caries. Complications such as a fracture, debonding, or the need for endodontic therapy were also analyzed. The Simplified Oral Hygiene Index (OHI‐S) was used to measure the patients' oral hygiene status (Greene & Vermillion, 1964). In this index, oral hygiene status is categorized based on the amount of plaque and calculus attached to the tooth surface. A disclosing solution was used to help detect retained plaque on the tooth surface. The oral hygiene status categories based on the OHI‐S measurements were good, fair, and poor oral hygiene.

### 2.1 Analysis

The interval survival rate of FDPs was analyzed by recording information concerning patient demographics. The patients were also clinically examined to register information related to the FDPs used, such as the type of prosthesis, prosthesis location, and type of complications associated with the prosthesis. Following that, the cumulative survival rate of FDPs from the time of treatment to the time of follow‐up was analyzed. Life‐table survival analyses were completed for FDP complications when secondary caries was present. The influence of oral hygiene status was evaluated using the nonparametric Kruskal–Wallis test. The Mann–Whitney *U* test was performed to test the significant differences between patients based on gender and material type. All data were statistically analyzed using the Statistical Package for the Social Sciences (SPSS) version 28 software (SPSS Inc.). The significance level was set at *p* ≤ .05.

## 3 RESULTS

A total of 423 patients were examined in this study, with a mean age of 43.7 years and a mean follow‐up time of 7 years. Of the patients, 39% were men (mean age of 46.1 years old and mean follow‐up time of 8.7 years), while 61% were women (mean age of 42.2 years old and mean follow‐up time of 6 years). A total of 1116 FDPs were evaluated, including 236 bridges, 863 crowns, and 67 veneer restorations. With regard to the materials used, 348 of the prostheses (31%) were made of full porcelain, while 768 (69%) were porcelain fused to metal (PFM).

Regarding complications, secondary caries was detected in 94 FDPs (8.4%), fracture (or chipping) in 85 (7.6%) cases, debonding in four (0.3%) cases, and the need for endodontic treatment in 42 cases (3.7%). These four complications (secondary caries, fracture/chipping, debonding, and the need for endodontic treatment) were seen in 213 of the 1116 FDPs (19%) examined in this study.

**Figure 1:**
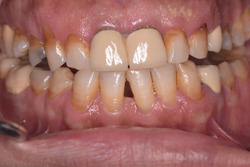
Secondary caries lesions at the margins of two connected porcelain fused to metal crowns on upper central incisors with poor plaque control.

**Figure 2:**
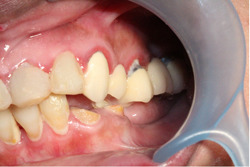
Female patient (37 years) presented to the clinic complaining of halitosis and periodontal inflammation related to the maxillary 5‐unit FPD, which was received 5 years ago. Secondary caries lesions can be seen around the margins of some teeth.

Furthermore, the occurrence of secondary caries was examined in relation to the patient oral hygiene status (Figures 1 and 2). Secondary caries was found in nine out of 219 (4%) patients with good oral hygiene, 35 out of 634 (5.5%) patients with fair oral hygiene, and 50 out of 272 (18.4%) patients with poor oral hygiene (*p* ≤ .001). Meanwhile, there was an obvious difference in the occurrence of secondary caries based on the patients' gender (*p* ≤ .001). In this study, 760 FDPs were placed in female patients, of which 42 had secondary caries (5.5%). For the male patients, 52 out of 356 prostheses (14.2%) were associated with secondary caries. As for the incidence of secondary caries based on the type of the material, full ceramic prostheses had a rate of 3.6% (13 out of 348), whereas PFM prostheses had a rate of 10.8% (83 out of 768; *p* = .142).

**Figure 3:**
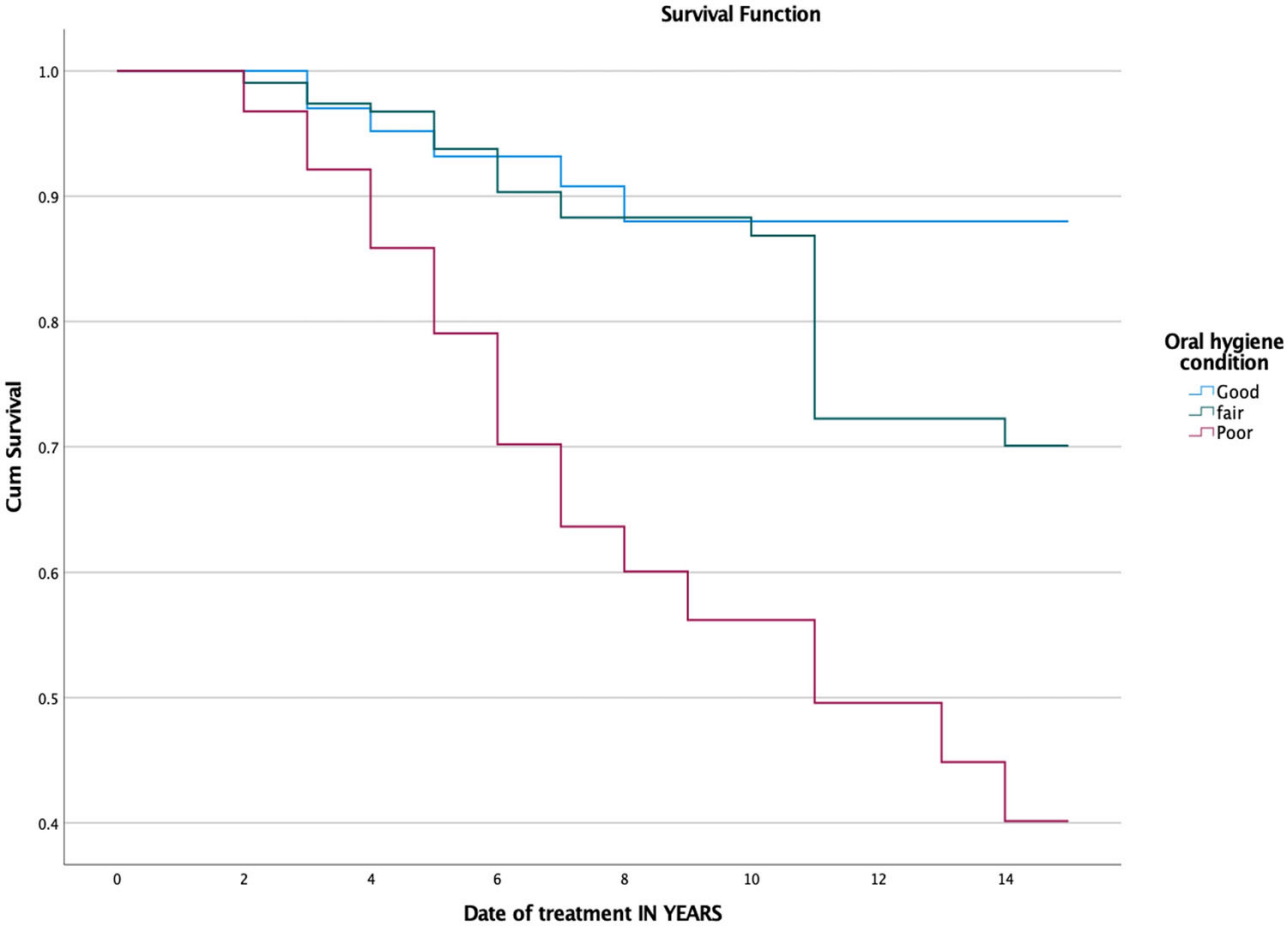
Kaplan–Meier survival function of FDPs for primary outcome “secondary caries” (*p* ≤ .001).

Based on the Kaplan–Meier method of estimation, there is an 88% probability that FDPs will survive secondary caries after 14 years in patients with good oral hygiene (Figure 3). The Kaplan–Meier analysis showed a lower probability of prosthesis survival in patients with fair and poor oral hygiene (70% and 40%, respectively). The cumulative survival rates of FDPs when secondary caries occurs in patients with different oral hygiene statuses are shown in Tables 1, 2, 3, 4.

**Table 1:** Life‐table survival analysis showing the cumulative survival rate of FDPs when it comes to the occurrence of secondary caries in all patients

Interval start time	Number entering interval	Number withdrawing during interval	Number exposed to risk	Number of recurrent caries	Proportion surviving (%)	Cumulative proportion surviving at the end of interval (%)	Std. error of (%)
0	423	0	423	0	100	100	0
1	423	64	391	5	99	99	1
2	354	46	331	9	97	96	1
3	299	45	276.5	7	97	94	1
4	247	15	239.5	10	96	90	2
5	222	32	206	10	95	85	2
6	180	18	171	7	96	82	2
7	155	18	146	3	98	80	3
8	134	14	127	2	98	79	3
9	118	4	116	1	99	78	3
10	113	19	103.5	12	88	69	3
11	82	3	80.5	0	100	69	3
12	79	4	77	2	97	67	4
13	73	1	72.5	3	96	65	4
14	69	1	68.5	0	100	65	4

Abbreviation: FDPs, fixed dental prostheses.

**Table 2:** Life‐table survival analysis showing the cumulative survival rate of FDPs when it comes to the occurrence of secondary caries in patients with good oral hygiene.

Interval start time	Number entering interval	Number withdrawing during interval	Number exposed to risk	Number of recurrent caries	Proportion surviving (%)	Cumulative proportion surviving at the end of interval (%)	Std. error of (%)
0	99	0	99.0	0	100	100	0
1	99	25	86.5	0	100	100	0
2	74	14	67.0	2	97	97	2
3	58	9	53.5	1	98	95	3
4	48	2	47.0	1	98	93	3
5	45	3	43.5	0	100	93	3
6	42	6	39.0	1	97	91	4
7	35	5	32.5	1	97	88	5
8	29	1	28.5	0	100	88	5
9	28	1	27.5	0	100	88	5
10	27	5	24.5	0	100	88	5
11	22	1	21.5	0	100	88	5
12	21	1	20.5	0	100	88	5
13	20	0	20.0	0	100	88	5
14	20	0	20.0	0	100	88	5

Abbreviation: FDPs, fixed dental prostheses.

**Table 3:** Life‐table survival analysis showing the cumulative survival rate of FDPs when it comes to the occurrence of secondary caries in patients with fair to moderate oral hygiene.

Interval start time	Number entering interval	Number withdrawing during interval	Number exposed to risk	Number of recurrent caries	Proportion surviving (%)	Cumulative proportion surviving at the end of interval (%)	Std. error (%)
0	227	0	227.0	0	100	100	0
1	227	31	211.5	2	99	99	1
2	194	27	180.5	3	98	97	1
3	164	29	149.5	1	99	97	1
4	134	9	129.5	4	97	94	2
5	121	24	109.0	4	96	90	3
6	93	8	89.0	2	98	88	3
7	83	10	78.0	0	100	88	3
8	73	11	67.5	0	100	88	3
9	62	2	61.0	1	98	87	3
10	59	11	53.5	9	83	72	5
11	39	2	38.0	0	100	72	5
12	37	3	35.5	0	100	72	5
13	34	1	33.5	1	97	70	5
14	32	1	31.5	0	100	70	5

Abbreviation: FDPs, fixed dental prostheses.

**Table 4:** Life‐table survival analysis showing the cumulative survival rate FDPs when it comes to the occurrence of secondary caries in patients with poor oral hygiene.

Interval start time	Number entering interval	Number withdrawing during interval	Number exposed to risk	Number of recurrent caries	Proportion surviving (%)	Cumulative proportion surviving at the end of interval (%)	Std. error (%)
0	97	0	97.0	0	100	100	0
1	97	8	93.0	3	97	97	2
2	86	5	83.5	4	95	92	3
3	77	7	73.5	5	93	86	4
4	65	4	63.0	5	92	79	5
5	56	5	53.5	6	89	70	5
6	45	4	43.0	4	91	64	6
7	37	3	35.5	2	94	60	6
8	32	2	31.0	2	94	56	6
9	28	1	27.5	0	100	56	6
10	27	3	25.5	3	88	50	7
11	21	0	21.0	0	100	50	7
12	21	0	21.0	2	90	45	7
13	19	0	19.0	2	89	40	7
14	17	0	17.0	0	100	40	7

Abbreviation: FDPs, fixed dental prostheses.

## 4 DISCUSSION

Numerous reports have described how FDPs can facilitate plaque accumulation, which carries great risks for caries and periodontitis (Ercoli & Caton, 2018; Srimaneepong et al., 2022). However, other studies have revealed no statistically significant difference between teeth with FDPs and control teeth with regard to plaque index levels (Spagnuolo et al., 2013; Valderhaug et al., 1993).

The null hypothesis of the present study was rejected as there was a strong relationship between oral hygiene and the occurrence of recurrent caries around FDPs (*p* ≤.001). These findings correspond with previous reports investigating the long‐term prognosis of FDPs (Abduo & Lyons, 2017). For example, Solá‐Ruíz and colleagues revealed in their study that secondary caries was the main reason for repairing FDPs after 7 years of follow‐up (Solá‐Ruíz et al., 2015). The findings of the present study showed an 8.4% incidence of secondary caries when considering all patients from different oral hygiene status groups after 14 years of follow‐up. However, several clinical studies have reported variations regarding the incidence of secondary caries, which ranges from 1.5% to 20% over long‐term observation periods (Raigrodski, 2004; Schmitt et al., 2012; Wolfart et al., 2009).

Also, patients with good oral hygiene were associated with a lower incidence of recurrent caries compared with patients with fair or poor oral hygiene (Tables 1, 2, 3). These results may indicate that oral hygiene conditions play an important role in the incidence of caries lesions in patients with or without FDPs. It has been suggested that the maintenance of good oral hygiene can reduce the risk of dental caries (John et al., 2017). However, some recently published reports showed that controlled trials did not find a strong correlation between good oral hygiene and a reduced risk of dental caries (Hujoel et al., 2018; Stein et al., 2018).

While this study did not investigate the time needed for the initiation of caries lesions, the survival analysis tests showed differences in the onset of caries between the patients with poor and fair oral hygiene. The onset of caries lesions, which is characterized by the demineralization process of the tooth surface, can occur soon after a tooth's eruption into the oral cavity (Baelum et al., 2003; Carvalho, 2014). In their experimental study, Thomas and colleagues found that secondary caries lesions appeared and progressed similarly to primary lesions (Thomas et al., 2007). Our results showed that there was a dramatic increase in the number of detected recurrent caries after 2 years of placing FDPs in patients with poor oral hygiene (Table 4). However, this dramatic increase came only after 10 years of follow‐up for patients with fair oral hygiene. Meanwhile, the 14‐year follow‐up of patients with good oral hygiene revealed a limited risk of recurrent caries. This can indicate that oral hygiene status has a great influence on the prognosis of dental treatment. This type of biological complication could be difficult to manage compared to some technical complications, such as chipping or debonding.

The treatment of secondary caries at the tooth–restoration interface may warrant different management approaches, such as new preparation margins, endodontic treatments, or tooth extraction (Sailer et al., 2007; Solá‐Ruíz et al., 2015). Thus, evaluating a patient's oral hygiene is a crucial part of developing a treatment plan. Patient motivation and oral hygiene care should be evaluated carefully before starting FDP treatment. Following treatment, the patient should be asked to continue their follow‐up visits to monitor the patient's oral hygiene status and detect any signs of complications with their FDPs.

The categorization of patients' oral hygiene status was based on the OHI‐S, which is a direct and simple index that focuses on measuring plaque and calculus retention on the tooth surface. However, while measuring plaque accumulation can provide information about plaque sites, it does not provide information on the history or rate of accumulation. This plaque accumulation record is a static measure and may not be suitable for evaluating patients' oral hygiene habits (Shilpa et al., 2019). Therefore, evaluating the plaque accumulation rate is believed to be a more proper method for assessing oral hygiene status and the risk of secondary caries (Marsh, 2006).

The evaluation of secondary caries in this study involved both clinical and radiographic examinations. These methods are typically used together to assess secondary caries around the margins, which can be confused with microleakage or staining when using visual inspection alone (Diniz et al., 2016; Nedeljkovic et al., 2015). Meanwhile, radiographs are known to be beneficial for assessing secondary caries affecting the margin and proximal contacts of FDPs. However, the radiopaque manifestation of FDPs under radiographs can sometimes hide the caries lesion completely or partially (Mjör, 2005). Thus, the literature demonstrates substantial variations among clinicians regarding the diagnosis and interpretation of recurrent caries (Clark & Mjör, 2001; Kidd, 2001).

Many researchers have investigated how FDP treatments impact oral hygiene status (Basnyat et al., 2015; Srimaneepong et al., 2022). It has been suggested previously that the construction design and type of materials used in the prostheses have a great influence on gingival health and plaque index (Spagnuolo et al., 2013). For instance, Srimaneepong colleagues revealed in their review that a fixed dental bridge has more influence on the plaque index compared with a crown restoration (Srimaneepong et al., 2022). Another review concluded that no differences can be found in the gingival health of crown restorations with a metal or metal‐ceramic composition (Nakamura et al., 2010). In addition to oral hygiene, it is believed that microleakage is a significant factor in the development of secondary caries (Land & Hopp, 2010). In fact, a clinical cohort study on fixed partial dentures reported the development of secondary caries in around 20% of cases because of marginal gaps (Sailer et al., 2007). However, many researchers have not supported this finding and have stressed that this solution can be applied only in large gaps exceeding 200 µm (Mjör & Toffenetti, 2000; Özer, 1997). Meanwhile, the presence of overhangs or over‐contoured crowns is considered a risk factor for patients to develop recurrent caries since it interrupts the self‐cleaning process and facilitates plaque accumulation (Gemalmaz & Ergin, 2002; Özer, 1997).

The authors of this paper stress that patients with FDPs require clear oral hygiene instructions. Patients should be aware of the difficulty of controlling plaque with FDPs compared with natural teeth. Patient education is also a crucial part of treatment using FDPs (Ahuja et al., 2016). One of the limitations of this study is that it did not investigate patients' histories of secondary caries. Also, the plaque index used to categorize the oral hygiene status was based on plaque accumulation, which is only one of the factors that describe the condition of patients' oral hygiene. Future investigations should consider other factors that may be associated with the occurrence of secondary caries.

## 5 CONCLUSION

The 10‐year estimated cumulative survival rate of FDPs was 69% when the occurrence of secondary caries treatment was considered as the main complication. Good oral hygiene has a great influence on reducing the risk of secondary caries in patients with FDPs. The occurrence of secondary caries is a common complication in patients with poor oral hygiene.

## AUTHOR CONTRIBUTIONS

Ali Alenezi contributed to conception, study design, data collection, analysis, and interpretation and drafted and critically revised the manuscript. Omar Alkhudhayri, Fahad Altowaijri, Lina Aloufi, Fatimah Alharbi, Maha Alrasheed, Hind Almutairi, Abdulltif Alanazi, Mohammed Yehya, and Dhafer Al Asmari contributed to the conception, data collection, and interpretation and critically revised the manuscript. All authors gave their final approval and agreed to be accountable for all aspects of the work.

## CONFLICT OF INTEREST

The authors declare no conflict of interest.

## Data Availability

Data available on request from the authors.
